# The Quack’s Paradise

**Published:** 1900-03

**Authors:** 


### TEXAS MEDICAL JOURNAL.

AUSTIN, TEXAS.

A MONTHLY JOURNAL OF MEDICINE AND SURGERY.

EDITED AND PUBLISHED BY
F. E. DANIEL, M. D., AND S. E. HUDSON, M. D.

Published Monthly at Austin, Texas, by Drs. Daniel and Hudson. Subscription price $1.00 a year in advance.

Eastern Representative: John Guy Monihan, St. Paul Building, 220 Broadway, New York City.

Official organ of the West Texas Medical Association, the Houston District Medical Association, the Austin District Medical Society, the Brazos Valley Medical Association, the Galveston County Medical Society, and several others.

### The Quack's Paradise.

**A DIPLOMA MILL IN TEXAS.**

As HAS been often stated in these columns, under the laws of Texas any persons—graduates in medicine or not—and in any number from three up, may call themselves a "Faculty," and for $10 purchase from the Secretary of State a charter to teach medicine. The charter does not stipulate how long, nor by what method (except it may teach by mail!), nor what kind of "medicine" is to be taught, nor does the State ask any questions whatever about the proposed "medical college." It does not require that there shall be any college or other building in the scheme, nor that the incorporators shall be graduates, or have a library, a lecture hall, a laboratory or even a dissecting room; these trifles cut no figure by the side of the ten dollars paid into the State treasury; and under the laws of Texas "diplomas" given, or sold, rather, by the incorporators of such a college are recognized as *license to practice medicine!* The only stipulation being that said diploma shall be registered in the office of the clerk of the district court where the "graduate" holding the diploma locates to practice. Some years ago "The Health Medical College" was "chartered," and alleged to be located at Mound City, Coryell county. There was no building,—nor any of the requisites of a medical college ever provided, nor was any semblance of teaching ever attempted, so far as we could ever ascertain. But some "diplomas" issued by this alleged college put in an appearance —and every requirement of the *law* having been fulfilled, the holders could not be molested. The Red Back brought the subject to the attention of Attorney-General Crane, and he directed that in every district where the "diplomas" of the "Health College" were on record the district attorney should institute criminal proceedings against the holders of such diplomas, alleging *fraud,* notwithstanding the strick compliance of the concern with the letter of the law. No case ever came to trial so far as I know, but the threat, it seemed, ran the "graduates" out of business and the incorporators out of the State.

In view of the foregoing facts, towit: that the State sells for $10 a charter "to teach medicine, by mail or otherwise," without any qualifying clause whatever, it is doubtful if the Attorney-General could have successfully prosecuted a suit against either the incorporators or those "practicing medicine" by virtue of the diploma of the alleged college. A "medical college" (one of the kind we are speaking of, would smell as bad by any other name) chartered by the State is "an accredited medical college" (see Webster), and Messrs. Ogden & Terrell's construction of the law in their opinion, which is being extensively circulated by this latest fake which the Secretary of State has chartered—the "New York Medical College," of San Antonio, is in accord with the recent decision of the Supreme Court of Texas (see TEXAS MEDICAL JOURNAL, November, 1899), and the diplomas, although sold outright for $50, are on a par with any other diploma—a *license to practice*! See announcement below. "Tuition, graduation and diploma, $50." There are no qualifications to this offer. "What the "tuition" will consist of is explained in the following remarkable correspondence. *Attendance* is not required, but is optional, and *graduation* is assured. Note, however, in the legal opinion given below, Messrs. Ogden & Terrell say that "a diploma issued after a regular course of instruction *in* said institution" is legal.

The "faculty" consists of a Dr. Forest, said to be a graduate of something somewhere,—his wife, not a graduate, and a brother, a non-graduate.

Now, how are these fellows to be gotten at? What will be done about it? Wait and see. The Red Back is "on to it," but does not intend to go off half-cocked. Wait.

The Legislature is wholly to blame for this state of things. For many years the medical profession and the TEXAS MEDICAL JOUR-

NAL have sought to show the law-makers the absurdity, the reckless danger, the menace to the public health of the whole State, of accepting as license to practice medicine a diploma from *anywhere*—even from State's own medical branch of our university, or from the best college in the world. "A diploma" is evidence of a degree having been conferred (Webster), and "conveys no rights or privileges whatever." Examination is the only test of qualification and fitness, and the State should apply it strictly and impartially to all who aspire to the privilege of practicing medicine—as other states do. A State Board of Medical Examiners is a *necessity* for the protection of the lives of our citizens—of tenfold more importance than *quarantine*.

For the text of the charter of the New York Medical College of San Antonio, and a timely and vigorous editorial on the subject, with other interesting matter connected with this outrage, see *Texas Medical News* (Austin, Texas) for February (ult.), a splendid piece of journalistic work upon which the Red Back cordially congratulates Brothers Denton & Smith.

The foregoing is an introductory to the subject matter—"sorter" like light soup or raw oysters before a square meal:

ANNOUNCEMENT.

OF THE

NEW YORK MEDICAL COLLEGE

OF SAN ANTONIO, TEXAS.

We base its claim to patronage, on scientific methods of teaching, which renders it possible to present the essential facts usually required in a 3 or 4 year course in a much shorter period. We present facts stripped of unnecessary theories. The College is chartered by Authority of State Legislature. The

*FREE CLINIC FOR THE BENEFIT OF THE POOR AS WELL AS FOR THE INSTRUCTION OF STUDENTS.*

Special attention is called to the Eye, Ear, Nose and Throat Clinic. Obstetrical cases attended FREE in all parts of the City.

Open All Day. Telephone 562.

Yours respectfully,
THE NEW YORK MEDICAL COLLEGE,
Address, 201 Avenue C.

DIRECTORS:

Drs. J. D. Forest, R. B. Forest, N. E. Forest.

### CORRESPONDENCE WITH THE COLLEGE.

LAREDO, TEXAS, February 12, 1900.

DEAR PROFESSORS: I saw in San Antonio Papers a advertisement about your Colledge. I can not efford to go to north, because it takes to long and costs to much money. I now do a little practise and I worked for 3 years in a drug store. I can fill nearly all Prescriptions and I got good success in my little practise and I beliefe I could do well if I would have a Diploama. I did not go to no school in this country yet, and that is the reason why I may be a little backwards in English. I want to know the quickest time what I can get a Diploama in because I have a Family and I have to make a living for them. I have a little money but not ennough to go to the north, so you can tell me all about it and the quickest time I can get trough. I learn verry quick, and I am accuainted with nearly all the poisons and the Drugs, and I can gess the weights nearly perfect so I know it would not take me long to get a Diploama. I want to knew if your Diploama is good in Laredo. please answer me soon, then I will take your lessons by mail or come if you do not keep me long. I will send the money right away. truly yours

FRITZ BLUTSTECHER.

P. O. Box 85.

---

[To the above letter the following classical production was duly received by "Mr. Blutstecher." Both are authentic; we have the original on file:]

REPLY.

Books needed cost $4.75.

San antonio, Tex., 2.14.1900.

Fritz Blutstecher

Box No. 85

Laredo Tex.

Dear sir:—

your letter to hand contents noted. glad to receive a letter from one who has the desire to elevate himself. now as to taking course in our institution will say the Price is $50.00 in advance with your knowledge of Drugs We can fix you out in Short time. You can study at Home for a short time then come and remain a short time and finish our Dieplomas are good in The entire state of Texas also in old Mexico arizona New Mexico Nevada Kansas, indian Territory Oklahoma Territory arkansas. and California

Enclosed find Announcement also a Legal Caption from Messrs

Ogden & Terrell attorneys who attend to our legal Business. stating our rights.

Trusting to hear from you and to be able to be of service to you we beg to remain Respt N. Y. M. C.

201 ave C San Antonio

---

[The above reply, which we have printed *verbatim*, was written on a note size sheet, upon the leaves of which was printed this "opinion":]

SAN ANTONIO, TEXAS, December 28th, 1899

The New York Medical College, City.

Gentlemen: Replying to your query in regard to the standing under the law of this State which graduates of your College will have, I beg, first to quote the following Article from our Penal Code:

"If any person shall practice for pay, or as a regular practitioner, medicine, in this State, in any of its branches or departments, or offer or attempt to practice without first having obtained a certificate of professional qualification from some authorized board of medical examiners, or without having a diploma from some 'accredited' medical college, chartered by the legislature of the state or *its authority,* in which the same is situated, he shall be punished by fine not less than fifty nor more than five hundred dollars."

While your College was not chartered by the State legislature, it necessarily was chartered by *its authority* or it could not have been chartered at all. As to the word "accredited," your College would certainly be accredited by some and perhaps discredited by others, as all colleges, no matter hom eminent, are. ["Accredited," as here used, simply means "authorized." See Webster.—ED.]

Art. 440 of the same Code makes it a penal offense, punishable as above, for any person to engage in the practice of medicine for pay, without having first filed for record in the office of the District Clerk of the County in which the person desires to practice a certificate from some authorized board of medical examiners or a diploma from some accredited medical college.

Now the advantage your College has over those chartered by the laws of other states is this: A graduate, having a diploma from your College, could enter upon the practice of his profession, without having obtained a certificate of professional qualification, and could not be punished under article 438, supra, while a person holding a diploma from some College, chartered by or under the laws of a sister State, would be compelled to obtain such a certificate or he would be subject to the fine therein mentioned. [Not so. A diploma from any State is on the same footing as one from Texas. —Ed.]

Very truly yours,

W. H. Lipscomb, Notary Public.

---

Tuition, graduation and diploma $50 for those enrolling at once.

New York Medical College,
201 Avenue C.

The following was enclosed with the reply:

### EXTRACT FROM LEGAL OPINION

### GIVEN BY OGDEN & TERRELL.

San Antonio, Texas, January 16th, 1900.

*The New York Medical College, City.*

Gentlemen: We have examined the certified copy from the office of the Secretary of State, of the State of Texas, of the Charter of New York Medical College, and it appears therefrom that this College was duly incorporated under the laws of the State of Texas, on the 5th day of December, 1899.

In reply to your inquiry as to whether the graduates of the New York Medical College holding diplomas therefrom issued to them *after regular course of instruction in said institution,* [Italics ours.—Ed.] have the lawful right to Practice Medicine in the State of Texas, without having a certificate from the Board of Medical Examiners, we beg to say it seems to be perfectly clear that graduates of that college may without violating any of the provisions of the laws of the State of Texas, practice their profession in any county in which they may cause said diploma to be duly recorded, without being required to obtain a certificate from the Board of Medical Examiners.

Yours truly,

Ogden & Terrell.

We respectfully refer anyone wishing further information as to the legal standing of the College to our Attorneys, Ogden & Terrell, Cor. Commerce and St. Mary's Sts., San Antonio, Texas.

New York Medical College,
201 Avenue C.

THAT $10,000 LIBRARY.—An announcement in a San Antonio paper some time ago, under bold headlines, stated that Mrs. Cupples, the widow of the late great surgeon and ex-president of the Texas State Medical Association, had "donated Dr. Cupples' library, valued at $10,000, to the 'New York Medical College,' of San Antonio, as a memento to perpetuate Dr. Cupples' name," or something to that effect.

Dr. Cupples left his family very poorly provided for, and Mrs. Cupples sought to sell the doctor's books and instruments to relieve pressing necessities. Intimate friends, leading physicians of San Antonio, who loved Dr. Cupples, some years ago made an inventory of his books and instruments at a fair valuation, most of them being very old and out of date, and it footed up $850, books and instruments. Some were sold off separately, and the balance have passed into the hands of the New York Medical College. It was not a $10,000 library, nor was it a gift. I have a letter from Mrs. Cupples in which after saying she was hard pressed for money she adds:

"I tried to sell [to others—ED.] and could do nothing, so I took it upon myself and sold the instruments and library to the school mentioned in your letter (N. Y. Med. Col., San Antonio). I sold part and gave some which I could get no price for."

The facts, as related to me by the above mentioned physicians, are: The college people bought the instruments and the best of the books for $200, part cash, part unsecured notes. While Mrs. Cupples was destroying the pamphlets, and perhaps some worthless books, the purchaser said in effect: "Don't throw them away, give them to us." She gave them to him, and that with "some book cases or shelves and some medicine and bandages" for which she could get nothing so she wrote me, is the extent of the "gift."

These statements are based on Mrs. Cupples letters to me, and on the verbal assurances of a physician of San Antonio, one of the most eminent and distinguished in the State, a former student of Dr. Cupples, and who holds in dear remembrance the many good, great and noble deeds, and the spotless character of the departed, dearly beloved Cupples. Dr. Cupples was an ethical gentleman, and would have scorned any attempt to identify him in any way with such an institution as this "College."

